# Assessment of tetralogy of Fallot–associated congenital extracardiac vascular anomalies in pediatric patients using low-dose dual-source computed tomography

**DOI:** 10.1186/s12872-017-0718-8

**Published:** 2017-12-04

**Authors:** Bi-yue Hu, Ke Shi, Yu-Ping Deng, Kai-Yue Diao, Hua-Yan Xu, Rui Li, Zhi-Gang Yang, Ying-Kun Guo

**Affiliations:** 1 Department of Radiology, West China Hospital, Sichuan University, 37# Guo Xue Xiang, Chengdu, Sichuan 610041 China; 2 Department of Radiology, The Medical Centre Hospital of Qionglai City, 172# Xinlin Road, Qionglai, Chengdu, Sichuan 611530 China; 3 Department of Radiology, West China Second University Hospital, Sichuan University, 20# Section 3 South Renmin Road, Chengdu, Sichuan 610041 China

**Keywords:** Tetralogy of Fallot, Dual-source computed tomography, Pediatrics, Radiation dosing, Extracardiac vascular anomalies

## Abstract

**Background:**

To investigate the diagnostic value of dual-source computed tomography (DSCT) in the evaluation of tetralogy of Fallot (TOF)-associated extracardiac vascular abnormalities in pediatric patients compared with transthoracic echocardiography (TTE).

**Methods:**

One hundred and twenty-three pediatric patients diagnosed with TOF were included in this retrospective study. All patients underwent DSCT and TTE preoperatively. All associated extracardiac vascular abnormalities and their percentages were recorded. The diagnostic performances of DSCT and TTE were compared based on the surgical results. The image quality of DSCT was rated, and the effective radiation dose (ED) was calculated.

**Results:**

A total of 159 associated extracardiac vascular deformities were confirmed by surgery. Patent ductus arteriosus (36, 22.64%), right-sided aortic arch (29, 18.24%), and pulmonary valve stenosis (23, 14.47%) were the most common associated extracardiac vascular abnormalities. DSCT was superior to TTE in demonstrating associated extracardiac anomalies (diagnostic accuracy: 99.13% vs. 97.39%; sensitivity: 92.45% vs. 77.07%; specificity: 99.81% vs. 99.42%). The agreement on grading the image quality of DSCT was excellent (κ = 0.80), and the mean score of the image quality was 3.39 ± 0.50. The mean ED of DSCT was 0.86 ± 0.47 mSv.

**Conclusions:**

Compared to TTE, low-dose DSCT has high diagnostic accuracy in the depiction of associated extracardiac vascular anomalies in pediatric patients with TOF, and could provide more morphological details for surgeons.

## Background

Tetralogy of Fallot (TOF) is the most common complex cyanotic congenital heart disease (CHD), with a prevalence of 3.5%. Almost all pediatric patients born with TOF can now expect to survive to adulthood because of advances in its surgical treatment [1]. Except for the four major malformations of TOF, precise associated extracardiac vascular anatomical information, particularly of the coronary and pulmonary arteries, is crucial for surgeons in the formulation of surgical strategies [2, 3]. Thus, effective imaging modalities are required to provide a thorough preoperative anatomic description of the associated extracardiac vascular anomalies in pediatric patients with TOF in order to improve surgical planning and outcomes [2–7].

In recent years, dual-source computed tomography (DSCT) has become regarded as a reliable non-invasive tool for delineating various anomalies in pediatric patients with complex CHD [3–5, 8–11]. To the best of our knowledge, few studies have focused on the preoperative evaluation of associated extracardiac vascular anomalies in pediatric patients with TOF using DSCT, especially not large case series studies. Therefore, in this study, we enrolled 123 pediatric patients to investigate the diagnostic performance of DSCT compared with that of transthoracic echocardiography (TTE) for assessing associated extracardiac vascular abnormalities in pediatric patients with TOF.

## Methods

### Study population

A total of 151 consecutive TOF patients who were referred to our hospital between June 2010 and September 2016 were enrolled. Exclusion criteria included non-surgical patients (*n* = 18), patients with unstable clinical conditions (*n* = 5), renal insufficiency (*n* = 2), and hypersensitivity to iodinated contrast (*n* = 3). Finally, 123 patients (69 males, 54 females; mean age: 2.85 ± 2.30 years; age range: from 5 months to 10 years; mean heart rate: 122.50 ± 16.73 bpm) were included. All patients underwent DSCT and TTE preoperatively. All associated extracardiac vascular abnormalities and their percentages were recorded. The study was approved by the Institutional Review Board at our institution (NO.14–163) who granted a waiver of patient consent due to the retrospective nature of the study.

### Scanning protocol

All examinations were performed using a DSCT scanner (SOMATOM Definition; Siemens Medical Solutions, Forchheim, Germany). Patients younger than 6 years of age usually underwent short-term sedation by an intravenous injection of chloral hydrate (concentration: 10%, dose: 0.5 ml/kg). The rest of the patients held their breath during scanning with full cooperation. The scans were performed from the inlet of the thorax to 2 cm below the diaphragm level in the craniocaudal direction. A nonionic contrast agent (iopamidol, 370 mg/ml; Bracco Sine Pharmaceutical Corp. Ltd., Shanghai, China), followed by 20 ml of saline solution, was injected into the antecubital vein at a rate of 1.2–2.5 ml/s and at a volume of 1.5 ml/kg body weight. With a predefined threshold of 100 HU, bolus tracking was used in the region of interest (ROI) in the descending aorta. Image acquisition was triggered when the ROI attenuation threshold reached 100 HU following a delay of 5 s. A retrospective ECG-gated protocol was used with the following acquisition parameters: tube current, 100 mAs; tube voltage, 80 kV; gantry rotation time, 0.28 s; and pitch, 0.2–0.5 (adjusted according to heart rate; higher heart rates used a higher pitch). The ECG pulsing window was set to auto.

All imaging data obtained were transferred to a workstation (Syngo; Siemens Medical Systems, Forchheim, Germany). Images were reconstructed with a slice thickness of 0.75 mm and increment of 0.7 mm. Two- and three-dimensional images were used for interpretation in all cases by means of maximum intensity projection, multiplanar reformation, and volume rendering.

### Imaging analysis

Two experienced radiologists analyzed each subject in a blind fashion. The four main characteristics of TOF [ventricular septal defect, overriding aorta, right ventricular outflow tract (RVOT) obstruction, and right ventricular hypertrophy] and associated extracardiac malformations were analyzed and recorded using a sequential segmental approach. When the radiologists disagreed on the findings, they discussed and reached a consensus before the surgical results were given.

To compare the diagnostic performance of DSCT with that of TTE, we categorized all the surgically confirmed associated extracardiac vascular deformities into five groups, namely abnormal vena cava connection (AVCC), aortic artery and valve disorders, anomalies of the pulmonary artery and valve, deformities of the aortopulmonary vessels, and coronary artery anomalies (CAAs).

### Assessment of image quality

The overall image quality of DSCT was rated on a four-point scale system in which 4 for excellent (excellent image quality, excellent visualization of anatomic structures), 3 for good (good image quality, clear delineation of anatomic details), 2 for fair (fair image quality, insufficient for complete evaluation), and 1 for poor (poor image quality, uninterpretable anatomic information with severe artifacts) [11]. Grades 3 or 4 were considered to be sufficient for diagnosis. Differences in opinion were resolved by discussion to achieve a consensus.

### Transthoracic echocardiography

TTE examinations were performed preoperatively in all patients using a Philips Sonos 7500 ultrasound system (Philips Medical Systems, Bothell, WA, USA) on the basis of the recommendations of the American Society of Echocardiography [12]. A skilled echocardiography practitioner not involved in the DSCT diagnosis interpreted the TTE images in a blind fashion.

### Radiation dose estimation

The volume CT dose index and dose–length product (DLP) were recorded during the examinations. The effective dose (ED) was derived from the DLP and a conversion coefficient *k* (ED = DLP × *k*), with infant-specific DLP conversion coefficients of 0.026 for patients between 4 months and 1 year of age, 0.018 for patients between 1 and 6 years of age, and 0.012 for patients between 6 and 10 years of age [13, 14].

### Statistical analysis

The data analysis was performed using SPSS software for Mac (version 24.0; IBM Corp., Armonk, NY, USA). Continuous variables were expressed as means ± standard deviations, and categorical variables were presented as numbers and percentages. The sensitivity, specificity, positive predictive value, and negative predictive value for DSCT and TTE were evaluated for the extracardiac anomalies in each group. Kappa values over 0.75, from 0.75 to 0.4, and below 0.4 were considered excellent, good to fair, and poor, respectively.

## Results

### Baseline characteristics

All patients underwent DSCT examinations without any complications. The mean age was 2.85 ± 2.30 years (range: 5 months to 10 years), the mean body mass index was 14.05 ± 6.48 kg/m^2^, and the mean heart rate was 122.50 ± 16.73 bpm. The most common symptoms in the pediatric patients referred to our hospital were heart murmurs (79/123, 64.23%), cyanosis (30/123, 24.39%), and post-exercise tachypnea (5/123, 4.07%) (Table 1).

**Table 1:** Baseline characteristics of pediatric patients with TOF

Variable	Total(*n* = 123)
Age, years	2.85 ± 2.30
Male, n (%)	69 (56%)
Body mass index (kg/m2)	14.05 ± 6.48
Heart rate (bpm)	122.50 ± 16.73
Systolic blood pressure (mmHg)	91.25 ± 24.84
Diastolic blood pressure (mmHg)	54.15 ± 18.68
Symptoms, n (%)	
Heart murmurs	79 (64.23%)
Cyanosis	30 (24.39%)
Squatting	2 (1.63%)
Post-exercising tachypnea	5 (4.07%)
Oppression in chest	1 (0.81%)
Medical history	
Brain abscess, n (%)	1 (0.81%)

Note: Data are presented as the number of patients (percentage) or as mean ± SD

### Comparison of diagnostic accuracy between DSCT and TTE

**Fig. 1:**
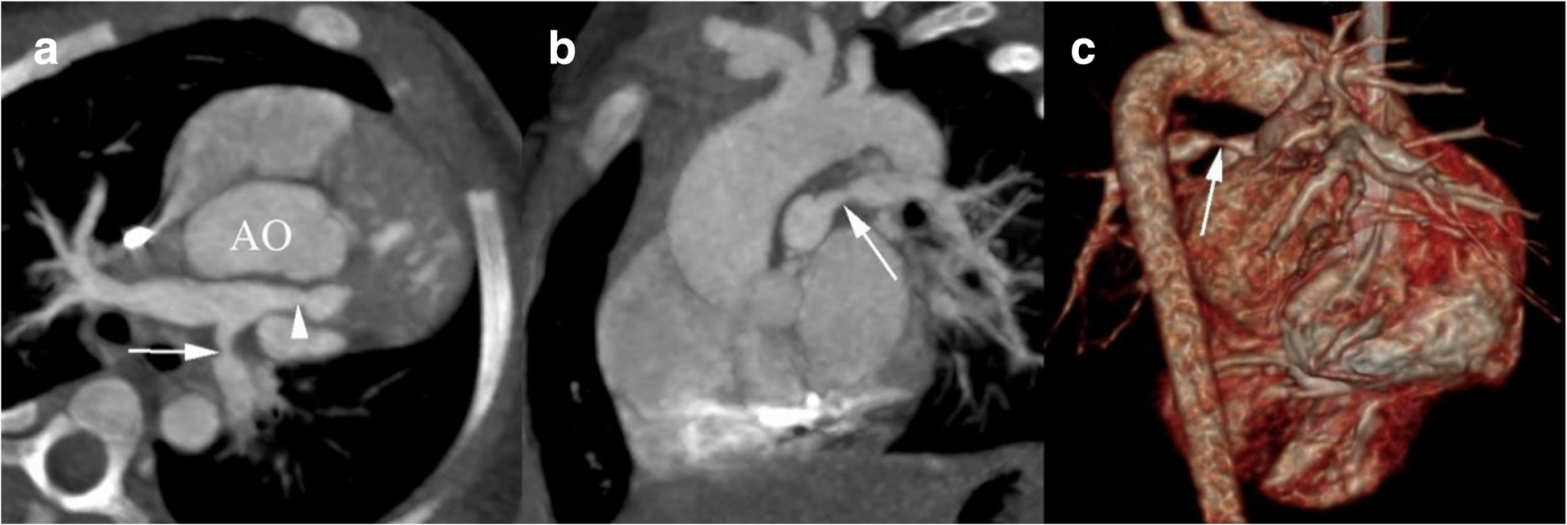
DSCT imaging in a 3 years old boy with TOF. **a** axial maximum intensity projection (MIP) image displays the major pulmonary artery stenosis (*arrow head*) and poor development of LPA (*arrow*). **b** The sagittal MIP image, and **c** The volume rendering image shows the poor development of LPA (*arrow*). AO, aorta; LPA, left pulmonary atery

**Fig. 2:**
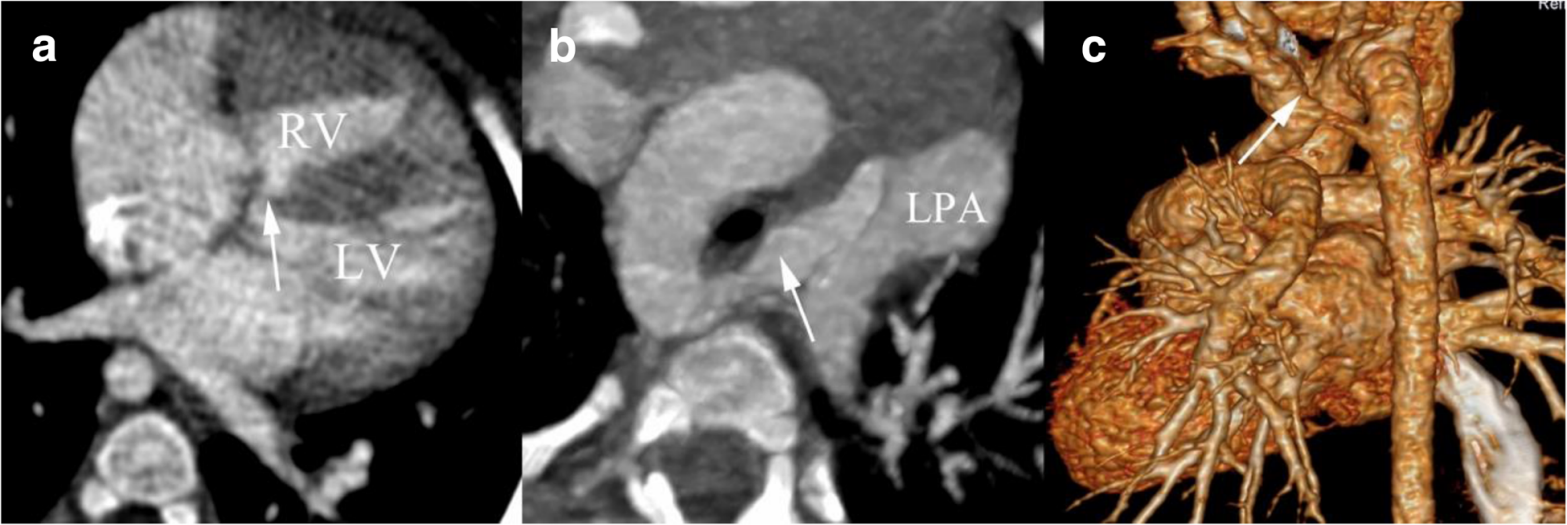
DSCT imaging in a 1 years old girl with TOF associated with double aortic arch with vascular ring formation. (**a**) axial image shows the tiny ventricular septal defect (*arrow*). **b** The axial maximum intensity projection image presents the patent ductus arteriosus between LPA and right-sided aortic arch (*arrow*). **c** The volume rendering image shows that double aortic arch with vascular ring formation (*arrow*). LPA, left pulmonary artery; LV, left ventricle; RV, right ventricle

**Fig. 3:**
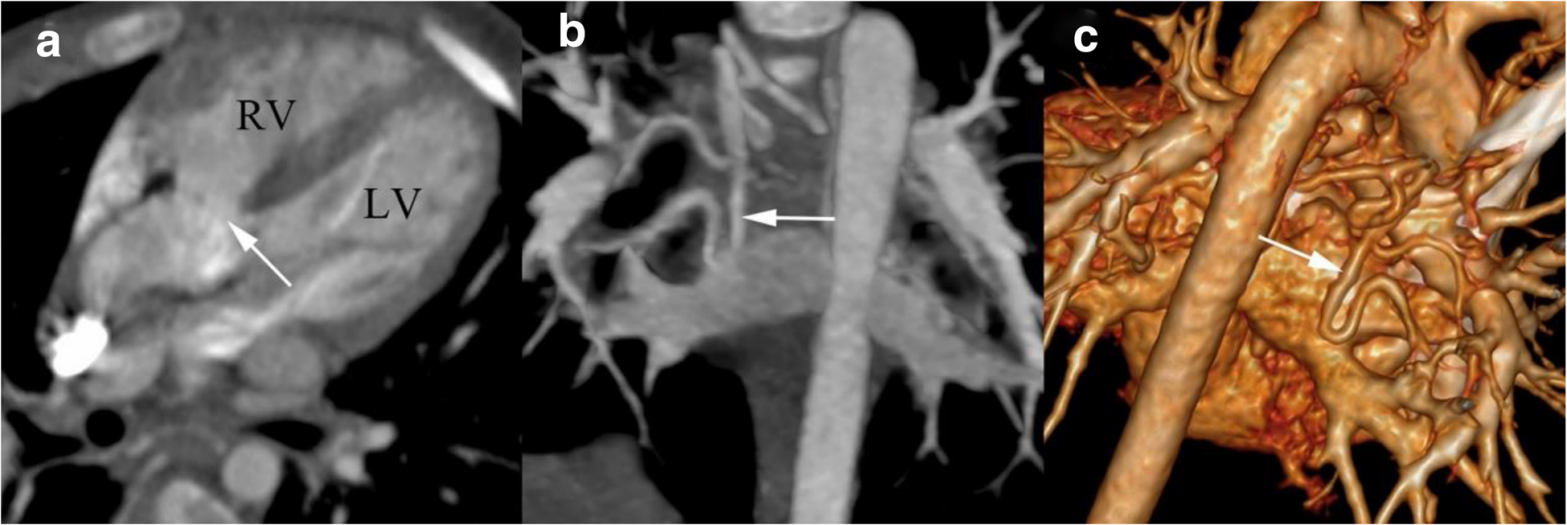
DSCT imaging in a 4 years old boy with TOF associated small aortopulmonary collateral vessels. **a** axial image shows the ventricular septal defect (*arrow*), over-riding of the aorta, and the small tortuous aortopulmonary collateral vessels (*arrowhead*). **b** The coronal maximum intensity projection (MIP) image and (**c**) The volume rendering (VR) image shows that small tortuous aortopulmonary collateral vessels (*arrow*). LV, left ventricle; RV, right ventricle

**Fig. 4:**
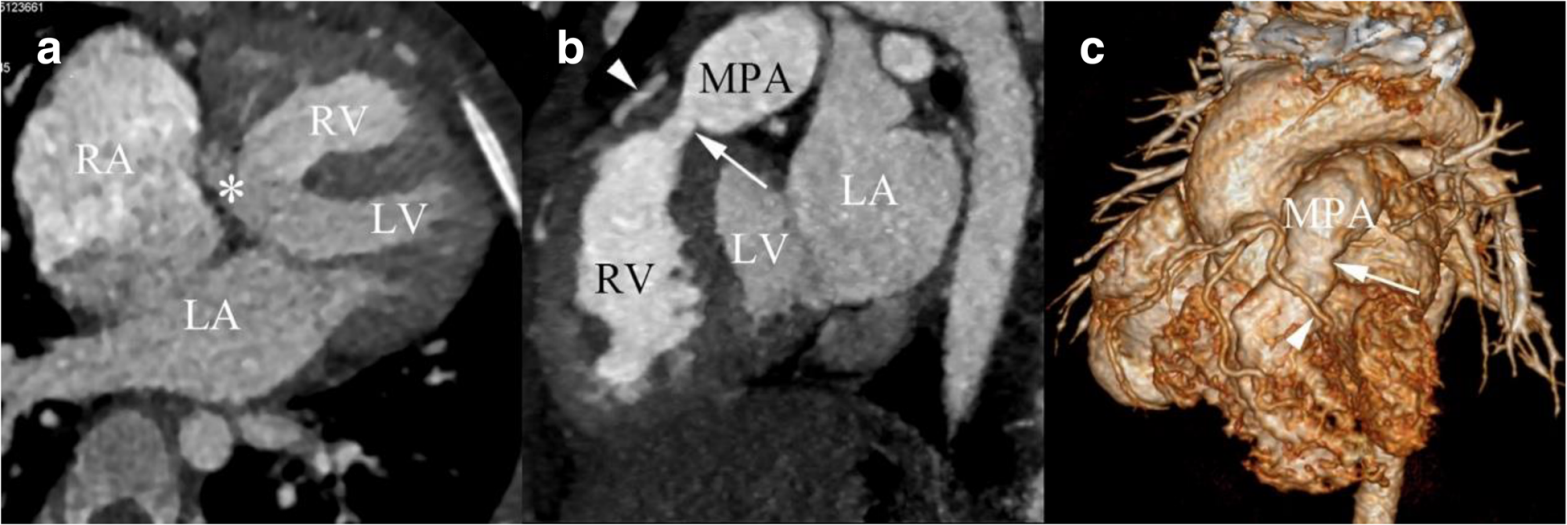
DSCT imaging in a 4 years old girl with TOF. **a** axial image shows the major malformations of TOF, including ventricular septal defect (*arrow*), over-riding of the aorta (asterisk), and right ventricular hypertrophy (*arrow head*). **b** The sagittal maximum intensity projection image presents the pulmonary artery stenosis (*arrow*) and the left anterior descending artery (LAD) crossing RVOT (*arrow head*). **c** The volume rendering (VR) image shows that the LAD (*arrow head*) arising from the right aortic sinus crossing RVOT and pulmonary artery stenosis (*arrow*). LA, left atrium; RA, right atrium; MPA, major pulmonary artery; LV, left ventricle; RV, right ventricle

DSCT was superior to TTE in demonstrating associated extracardiac vascular anomalies (diagnostic accuracy: 99.13% vs. 97.39%; sensitivity: 92.45% vs. 77.07%; specificity: 99.81% vs. 99.42%; positive predictive value: 98.00% vs. 93.08%; negative predictive value: 99.24% vs. 97.74%). Twelve associated extracardiac vascular anomalies were not detected by DSCT, including 1 case of anomalous hepatic venous connection, 2 of bicuspid aortic valve, 1 of pulmonary valve stenosis, 2 of bicuspid pulmonary valve, 1 of quadricuspid pulmonary valve, 1 of pulmonary atresia, 2 of absent pulmonary valve syndrome, and 2 of patent ductus arteriosus (PDA). Conversely, TTE missed 36 associated extracardiac vascular anomalies, including 4 cases of persistent left superior vena cava, 1 of anomalous hepatic venous connection, 10 of right-sided aortic arch, 1 of double aortic arch, 4 of supravalvular pulmonary stenosis (Fig. 1), 1 of quadricuspid pulmonary valve, 1 of pulmonary atresia, 2 of absent pulmonary valve syndrome, 6 of PDA (Fig. 2), 4 of aortopulmonary collateral vessels (Fig. 3), and 2 of CAAs (Fig. 4). DSCT misdiagnosed 1 patient with bicuspid pulmonary valve and 2 with PDA, whereas TTE misdiagnosed 1 patient with right-sided aortic arch, 3 with PDA, 2 with aortopulmonary collateral vessels, and 3 with CAAs (Table 2).

**Table 2:** A summary of the associated extra-cardiac vascular deformities by DSCT and TTE (*n* = 123)

Extra-cardiac vascular anomalies	Surgical results	DSCT findings				TTE findings			
		TP	FN	TN	FP	TP	FN	TN	FP
Abnormal vena cava connection									
Persistent left superior vena cava	18 (11.32%)	18	0	105	0	14	4	105	0
Anomalous hepatic venous connection	1 (0.63%)	0	1	122	0	0	1	122	0
Aortic artery and valve disorders									
Right-sided aortic arch	29 (18.24%)	29	0	94	0	19	10	93	1
Double aortic arch	1 (0.63%)	1	0	122	0	0	1	122	0
Bicuspid aortic valve	2 (1.26%)	0	2	121	0	2	0	121	0
Anomalies of the pulmonary artery and valve									
Supravalvular pulmonary stenosis	15 (9.43%)	15	0	108	0	11	4	108	0
Pulmonary valve stenosis	23 (14.47%)	22	1	100	0	23	0	100	0
Bicuspid pulmonary valve	5 (3.14%)	3	2	117	1	5	0	118	0
Quadricuspid pulmonary valve	1 (0.63%)	0	1	122	0	0	1	122	0
Pulmonary atresia	2 (1.26%)	1	1	121	0	1	1	121	0
Absent pulmonary valve syndrome	2 (1.26%)	0	2	121	0	0	2	121	0
Deformities of the aortopulmonary vessels									
Patent ductus arteriosus	36 (22.64%)	34	2	85	2	28	6	86	3
Aortopulmonary collateral vessels	22 (13.84%)	22	0	101	0	18	4	99	2
Coronary artery anomalies	2 (1.26%)	2	0	121	0	0	2	118	3

*Abbreviations: TP* true positive finding, *FN* false negative finding, *TN* true negative finding, *FP* false positive finding

The diagnostic performance of DSCT for each group was superior to that of TTE (sensitivity for instance: AVCC: DSCT, 94.74% vs. TTE, 73.68%; aortic artery and valve disorders: DSCT, 93.75% vs. TTE, 65.63%; anomalies of pulmonary artery and valve: DSCT, 85.42% vs. TTE, 83.33%; deformities of aortopulmonary vessels: DSCT, 96.55% vs. TTE, 82.14%; CAAs: DSCT, 100% vs. TTE, 0%) (Table 3).

**Table 3:** The diagnostic accuracy of DSCT and TTE (*n* = 123)

Anomalies categories	DSCT				TTE			
	sen	spec	ppv	npv	sen	spec	ppv	npv
Abnormal vena cava connection	94.74%	100%	100%	99.56%	73.68%	100%	100%	97.84%
Aortic artery and valve disorders	93.75%	100%	100%	99.41%	65.63%	99.70%	95.45%	96.83%
Anomalies of the pulmonary artery and valve	85.42%	99.86%	97.62%	98.99%	83.33%	100%	100%	98.85%
Deformities of the aortopulmonary vessels	96.55%	98.94%	96.55%	98.94%	82.14%	97.37%	90.20%	94.87%
Coronary artery anomalies	100%	100%	100%	100%	0.00%	97.52%	0.00%	98.33%

*Abbreviations: Sen* sensitivity, *Spec* specificity, *PPV* positive predictive value, *NPV* negative predictive value

### Image quality assessment

Diagnostic DSCT images (images with scores of 3 or 4) were obtained in all examinations. The mean subjective image quality score of the 123 cases was 3.39 ± 0.50, and the score distribution was as follows: 3 (*n* = 75, 61%) and 4 (*n* = 48, 39%). The interobserver agreement on the overall image quality scoring (κ = 0.80) indicated excellent agreement.

### Radiation dose estimation

The mean DLP for patients between 4 months and 1 year of age was 39.50 ± 19.20 mGy.cm, and the estimated mean ED was 1.03 ± 0.50 mSv. The mean DLP for patients between 1 and 6 years of age was 40.80 ± 18.80 mGy.cm, which corresponds to an estimated mean ED of 0.73 ± 0.34 mSv. The mean DLP for patients between 6 and 10 years of age was 37.00 ± 16.82 mGy.cm, resulting in an estimated mean ED of 0.44 ± 0.20 mSv. The mean ED of all the subjects was 0.86 ± 0.47 mSv (Table 4).

**Table 4:** Radiation dose estimation adjusted to different age groups

	4 months to 1 years	1 years to 6 years	6 years to 10 years
CTDIvol (mGy)	7.79 ± 3.95	8.61 ± 6.06	8.69 ± 5.55
DLP (mGy.cm)	39.50 ± 19.20	40.80 ± 18.80	37.00 ± 16.82
ED (mSv)	1.03 ± 0.50	0.73 ± 0.34	0.44 ± 0.20

Abbreviations: *CTDIvol*, volume CT dose index; *DLP*, dose-length product; *ED*, effective dose

## Discussion

There is a high incidence of associated extracardiac vascular anomalies in pediatric patients with TOF, and these abnormalities might preclude certain types of surgical repair and may provoke late adverse outcomes [3, 6, 7]. Thus, imaging plays a key role in the precise preoperative evaluation of these associated extracardiac vascular anomalies in pediatric patients with TOF [2, 4].

TTE is the first-line option for depicting complex CHD. Combined with Doppler flow imaging, TTE is preferred in the diagnosis of intracardiac anomalies. However, its small acoustic window, low spatial resolution, and operator-dependent nature are inherent limitations that affect its diagnostic performance in identifying extracardiac vascular anomalies [4, 9]. Although cardiac catheterization (CCA) has served as the gold standard for cardiac imaging with hemodynamic evaluation, its high radiation dose and catheter-related complications from its invasive nature are the major deterrents of using this tool [15]. Magnetic resonance imaging (MRI) without X-ray exposure has been considered a promising imaging modality in recent years. Nevertheless, contraindications such as pacemakers, the need for lengthy sedation, and relatively lower spatial resolution limit the use of MRI in assessing the smaller extracardiac vascular deformities, particularly CAAs [16]. DSCT, with its rapid acquisition speed, high spatial and temporal resolution, and powerful image post-processing techniques, is rapidly becoming one of the most valuable modalities for cardiovascular examination [8–11].

In our study, compared with TTE, DSCT was of greater value in the visualization of AVCC and aortic artery and valve disorders; it was also better at detecting anomalies of the pulmonary artery and valve, as well as deformities of the aortopulmonary vessels. The small field of view during examination from the suprasternal direction could be the main reason why the great vessels could not be identified as accurately using TTE than with DSCT. Moreover, the short neck of pediatric patients, the overlying bone, and the aerated lung might also influence the diagnostic value of TTE in the depiction of these deformities. Regarding aortopulmonary collateral vessels (APCVs), TTE can only identify relatively large ones, whereas the number, origin, and supplied lung lobes of APCVs could be visualized on DSCT regardless of the size of the vessels [9, 17]. Furthermore, DSCT could provide an intuitive view of APCVs and their relationships with the large airways, which could help in surgical planning [7]. In terms of valvular anomalies, DSCT missed two cases bicuspid pulmonary valve, whereas one case of quadricuspid pulmonary valve and two cases of absent pulmonary valve syndrome were not depicted by either approach. This could be partially explained by the fact that DSCT imaging requires the transfer of digital information to gray-scale images, and therefore, some valvular details may be too small to be visualized [11]. Although TTE combined with color Doppler flow imaging and dynamic functional sequels has advantages over DSCT in the depiction of some valvular anomalies, various complex and rare valvular abnormalities such as quadricuspid pulmonary valve may not be able to be confirmed by either modality.

In addition, accurate preoperative detection of CAAs is essential for patients with TOF, because right ventriculotomy is required to relieve RVOT obstruction during the surgical correction of TOF [3, 8]. However, any major coronary artery that crosses the RVOT, such as the left anterior descending artery, could be accidentally damaged during the surgery. Our results showed that DSCT can detect this disorder in TOF patients with 100% sensitivity and 100% specificity, which is in agreement with the results of a previous study [3]. The relationship to great arteries, ostia number and location, and the length of the coronary arteries that can be seen and evaluated accurately through the analysis of axial and three-dimensional reconstructed images by DSCT [18]. Preoperative DSCT may thus help to optimize the surgical procedure to preserve the anomalous artery and improve the patients’ outcomes.

On the whole, DSCT has fine diagnostic performance in the preoperative assessment of associated extracardiac vascular malformations in pediatric patients with TOF. However, it can still miss some small and rare valvular malformations. Therefore, combining the findings of DSCT and TTE may be beneficial to improve the diagnostic accuracy of DSCT for these deformities.

The risk of radiation exposure remains a major concern for the pediatric population with CHD undergoing DSCT, especially during infancy. These risks cannot be ignored given the increased rate of DNA mutations and higher lifetime attributable risk for fatal and nonfatal cancers in these pediatric patients [19, 20]. Thus, DSCT was performed strictly in accordance with the ALARA (as low as reasonably achievable) principle in the present study. It is worth noting that several measures were applied to minimize the estimated mean ED to < 1 mSv (0.86 ± 0.47 mSv), which is much lower than that of CCA (approximately 4.6 mSv) [21, 22], including decreasing the tube voltage to the low level of 80 kV, applying the heart rhythm adaptive pitch, and using ECG-based tube current modulation as shown in previous studies [23–25].

The present study has several limitations. First, DSCT scans expose pediatric patients to radiation. Thus, we took several effective measures to minimize the radiation dose; the mean ED was 0.86 ± 0.47 mSv, which is much lower than that of CCA. Second, we did not identify the postoperative characteristics and outcomes with a long-term follow-up; this will be discussed in a future study. Finally, this is a retrospective single-center study, and larger, multicenter studies are therefore required in future.

## Conclusion

In summary, low-dose DSCT is superior to TTE for detecting extracardiac vascular anomalies in pediatric patients with complex CHD such as TOF. As a complementary modality of TTE, it offers comprehensive anatomical information on extracardiac vascular lesions in fine detail as part of the surgical preparation for TOF.

## Abbreviations

APCVs: Aortopulmonary collateral vessels
AVCC: Abnormal vena cava connection
CAAs: Coronary artery anomalies
CHD: Cyanotic congenital heart disease
DLP: Dose–length product
DSCT: Dual-source computed tomography
ED: Radiation dose
MRI: Magnetic resonance imaging
PDA: Patent ductus arteriosus
ROI: Region of interest
RVOT: Right ventricular outflow tract
TOF: Tetralogy of Fallot
TTE: Transthoracic echocardiography

## References

[1] Apitz C, Webb GD, Redington AN (2009). Tetralogy of Fallot. Lancet.

[2] Meinel FG, Huda W, Schoepf UJ, Rao AG, Cho YJ, Baker GH (2013). Diagnostic accuracy of CT angiography in infants with tetralogy of Fallot with pulmonary atresia and major aortopulmonary collateral arteries. J Cardiovasc Comput Tomogr.

[3] Vastel-Amzallag C, Le Bret E, Paul JF, Lambert V, Rohnean A, El Fassy E (2011). Diagnostic accuracy of dual-source multislice computed tomographic analysis for the preoperative detection of coronary artery anomalies in 100 patients with tetralogy of Fallot. J Thorac Cardiovasc Surg.

[4] Shi K, Yang ZG, HY X, Zhao SX, Liu X, Guo YK (2016). Dual-source computed tomography for evaluating pulmonary artery in pediatric patients with cyanotic congenital heart disease: comparison with transthoracic echocardiography. Eur J Radiol.

[5] Gao Y, Lu B, Hou Z, Yu F, Cao H, Han L (2012). Low dose dual-source CT angiography in infants with complex congenital heart disease: a randomized study. Eur J Radiol.

[6] Siripornpitak S, Pornkul R, Khowsathit P, Layangool T, Promphan W, Pongpanich B, Cardiac CT (2013). Angiography in children with congenital heart disease. Eur J Radiol.

[7] Dillman JR, Hernandez RJ (2009). Role of CT in the evaluation of congenital cardiovascular disease in children. AJR Am J Roentgenol.

[8] Shi K, Gao HL, Yang ZG, Zhang Q, Liu X, Guo YK (2017). Preoperative evaluation of coronary artery fistula using dual-source computed tomography. Int J Cardiol.

[9] Nie P, Yang G, Wang X, Duan Y, Xu W, Li H (2014). Application of prospective ECG-gated high-pitch 128-slice dual-source CT angiography in the diagnosis of congenital extracardiac vascular anomalies in infants and children. PLoS One.

[10] Opolski MP, Pregowski J, Kruk M, Staruch AD, Witkowski A, Demkow M (2014). The prevalence and characteristics of intra-atrial right coronary artery anomaly in 9,284 patients referred for coronary computed tomography angiography. Eur J Radiol.

[11] Shi K, Yang ZG, Chen J, Zhang G, HY X, Guo YK (2015). Assessment of double outlet right ventricle associated with multiple malformations in pediatric patients using retrospective ECG-gated dual-source computed tomography. PLoS One.

[12] Campbell RM, Douglas PS, Eidem BW, Lai WW, Lopez L, Sachdeva R (2014). ACC/AAP/AHA/ASE/HRS/SCAI/SCCT/SCMR/SOPE 2014 appropriate use criteria for initial transthoracic echocardiography in outpatient pediatric cardiology: a report of the American College of Cardiology Appropriate use Criteria Task Force, American Academy of Pediatrics, American Heart Association, American Society of Echocardiography, Heart Rhythm Society, Society for Cardiovascular Angiography and Interventions, Society of Cardiovascular Computed Tomography, Society for Cardiovascular Magnetic Resonance, and Society of Pediatric Echocardiography. J Am Coll Cardiol.

[13] Cousins C, Miller DL, Bernardi G, Rehani MM, Schofield P, Vañó E (2013). International Commission on Radiological Protection. ICRP PUBLICATION 120: radiological protection in cardiology. Ann ICRP.

[14] Deak PD, Smal Y, Kalender WA, Multisection CT (2010). Protocols: sex- and age-specific conversion factors used to determine effective dose from dose-length product. Radiology.

[15] Feltes TF, Bacha E, Beekman RH 3rd, Cheatham JP, Feinstein JA, Gomes AS, et al; American Heart Association congenital cardiac defects Committee of the Council on cardiovascular disease in the young; Council on Clinical Cardiology;Council on Cardiovascular Radiology and Intervention; American Heart Association. Indications for cardiac catheterization and intervention in pediatric cardiac disease: a scientific statement from the American Heart Association. Circulation. 2011;123(22):2607-52. 10.1161/CIR.0b013e31821b1f10 21536996

[16] Tangcharoen T, Bell A, Hegde S, Hussain T, Beerbaum P, Schaeffter T (2011). Detection of coronary artery anomalies in infants and young children with congenital heart disease by using MR imaging. Radiology.

[17] Chandrashekhar G, Sodhi KS, Saxena AK, Rohit MK, Khandelwal N (2012). Correlation of 64 row MDCT, echocardiography and cardiac catheterization angiography in assessment of pulmonary arterial anatomy in children with cyanotic congenital heart disease. Eur J Radiol.

[18] Cook SC, Raman SV (2007). Unique application of multislice computed tomography in adults with congenital heart disease. Int J Cardiol.

[19] Beauséjour Ladouceur V, Lawler PR, Gurvitz M, Pilote L, Eisenberg MJ, Ionescu-Ittu R (2016). Exposure to low-dose ionizing radiation from cardiac procedures in patients with congenital heart disease: 15-year data from a population-based longitudinal cohort. Circulation.

[20] Johnson JN, Hornik CP, Li JS, Benjamin DK, Yoshizumi TT, Reiman RE (2014). Cumulative radiation exposure and cancer risk estimation in children with heart disease. Circulation.

[21] Coles DR, Smail MA, Negus IS, Wilde P, Oberhoff M, Karsch KR (2006). Comparison of radiation doses from multislice computed tomography coronary angiography and conventional diagnostic angiography. J Am Coll Cardiol.

[22] Garg N, Walia R, Neyaz Z, Kumar S (2015). Computed tomographic versus catheterization angiography in tetralogy of Fallot. Asian Cardiovasc Thorac Ann.

[23] Faggioni L, Neri E, Sbragia P, Pascale R, D'Errico L, Caramella D, et al. 80-kV pulmonary CT angiography with 40 mL of iodinated contrast material in lean patients: comparison of vascular enhancement with iodixanol (320 mg I/mL) and iomeprol (400 mg I/mL). AJR Am J Roentgenol 2012;199(6):1220-1225. 10.2214/AJR.11.8122 23169711

[24] McCollough CH, Primak AN, Saba O, Bruder H, Stierstorfer K, Raupach R (2007). Dose performance of a 64-channel dual-source CT scanner. Radiology.

[25] Stolzmann P, Scheffel H, Schertler T, Frauenfelder T, Leschka S, Husmann L (2008). Radiation dose estimates in dual-source computed tomography coronary angiography. Eur Radiol.

